# Philadelphia County Medical Society

**Published:** 1891-09

**Authors:** 


### Society Proceedings.

### PHILADELPHIA COUNTY MEDICAL SOCIETY.

*Stated Meeting, May 27, 1891.*

THE Vice-President, DE FOREST WILLARD, M. D., in the chair.

Dr. JOSEPH HOFFMAN submitted a paper on

### THE RELATION OF IMPERFECT SURGERY TO THE SEQUELÆ OF PELVIC AND ABDOMINAL OPERATIONS.

So much has lately been said and written relative to the results and, therefore, to the justifiability of abdominal and pelvic operations, that it is necessary for the candid critic and honest operator to stop and consider what, on the one hand, is the exact status of the repentant critics and of their changed attitude toward the position and merits of surgery, and on the other how far inexact methods are accountable for some of the failures and misfortunes of what would otherwise be a field of almost unprecedented brilliancy and encouragement, in the results that have been obtained in this branch of surgery.

In the first place it is to be noted that of all the men who have gained prominence in any of the various domains of surgery in general, not one has abandoned that specialty from a surgical standpoint in order to treat it empirically by any other method. Sir Henry Thompson, for instance, has not departed from the exact surgery of the bladder in order to destroy its calculi by solvents or electricity; Mr. Macewen, bone surgery; or Shaffer, orthopedics. What is meant to be here illustrated is, that when a man has once chosen a specialty and worked up to that specialty by an experience gained by natural selection and application, his specialty has become so much a part of him that its abandonment is just as impossible as the negation of the laws of gravity. His training makes it the law of his mental gravity, and the same laws apply to it as to weight and inertia in the physical world. The greater his experience, the longer his training, the more certain is he in regard to the limitations, the requirements, the possibilities of his work, and as a result of all this his position, when taken, is an entrenched one, from which he is not to be moved and made a weathercock of every shade of opinion, whether of madmen, fools or philosophers; for none of these are apt to speak from a practical experience, in surgery at least; and practice, not theory, has made surgery what it is.

That there are specialties in surgery has come about by the consensus of opinion among surgeons in general, and physicians, strictly so-called, that there is need of them. The specialist in any branch is the living embodiment of the necessity of his work. To whom then is the title of "specialist" to be applied? In each branch confessedly, to those who work conformably with their expressed sentiment; or if not, teachers whose line of procedure is uniform and the result either of their own experience wrought out by laborious painstaking, or conforms with that of other expert workers in the same line.

To that class of see-saw workers who anon are this and anon are that, the name specialist should not be applied. For accurate, exact surgery, we cannot look to the electrician or the dabbler in it. If one condemns a procedure today and extols it tomorrow, we are brought to the point of inquiry, Which view is correct, which has the vantage of observation ? or perhaps, Is either expression of any value whatever ? or, Is the change made like that of the sleight-of-hand man to puzzle his audience and bring in a set of new admirers to be pleased by the rare and startling exhibition of surgical acrobatics,—a surgery in which the constant is differentiated out of sight by the variable ?

These inquiries and lines of thought are suggestive of some of the causes we have to consider in the relation of imperfect work to the sequelæ of abdominal and pelvic surgery. What has been said of experience as a necessity for a firm faith in the necessity of any special branch of work, is true with just as much force when the ability to do such work is considered. The appalling eagerness with which men with only a diploma as a justification and a safeguard from the hand of the law to do abdominal surgery, is one of the startling features in the history of this work. In no other branch of the art has there ever been any approach to such audacity. That fools rush in where angels fear to tread has never been more aptly illustrated. Training has been,—nay still is—rejected, while the work is sought, and if the case is found willing to be offered up an offering to the prurient itch of a surgical pretender, the case is at once reported as a wonderful cure in the hands of the youthful aspirant; or if unsuccessful is recorded, heralded, and posted up as a warning against all surgery in general, and against that surgeon in particular who has been rash enough to loan both his instruments and experience to a neophyte, with no other experience than in-grown toe-nails and vaccination, now hiding and excusing him self and his failure behind the experience of the too easy friend, who by a mistaken kindness has martyred both himself and surgery, and done both an irreparable injury. I take it that it should be the rule of all surgeons to assist no one who has not studied, observed and assisted in such work. There is no excuse or reason here for men to begin with all the faults and errors of the early operators, and again work out the technique of this branch of surgery in a series of failures and calamities that once well-nigh led to its abandonment. The work of such men cannot be other than imperfect, and must reflect upon surgical interference in these affections in the minds of the uncritical and unthinking. Another class of operators to be carefully watched, is one which, by fair means or foul, is bound to attain prominence. These men have lain in wait for operations, as the moonshiner for a cloudy night, to bring themselves into prominence, let us say notoriety. These men have opened abdomens, not to operate, but to do the first step of an operation. I can this moment put my hands on such a case, in whom nothing was found wrong, but there was a chance to perpetrate a pet fad, and the woman is now dragging out an existence after three subsequent operations, all the result of the first unnecessary tinkering. If we are to criticise abdominal surgery, let us not do so with the results of such operators and their methods before our eyes. Let us rather criticise the crude methods and cruder morals, or, if you please, the code that tolerates apprentices and dabblers to do with the bodies of our patients the like of which would ruin the reputation of our tailor, or well-nigh mob a cobbler. That such surgery has been overdone is just as true as it is that it has no right to be considered surgery at all. And just here it is to be said that among men of this class, I mean those who have done the kind of work just referred to, we are bound to find our latter-day conservatives and repentants. Of all those who have gained and kept a name as foremost in the rank of abdominal surgeons, we find no recanters. These are almost always to be found in the lines of failure, or discouragement, or embarrassment. If a man has made blunders, he is a fool not to perceive them, or if the real spirit of surgery is beyond him, and he feels it, he alike is bound on the one hand to retrieve past misfortune by so-called conservatism, sitting on the *pons asinorum* watching the stream of surgery flow by, and with it the hopes that he no more has chance of realizing. On the other, having neither the spirit of surgery within him, nor the courage to attain it by drill and application, nothing is left for him but to be a dissenter, to decry all surgery as mutilation, nothing justifying but conservatism. From this standpoint I make the plea that each one of us, before he condemns surgery, its justifiability or its results, shall judge of them all. As sensible men and women, saying that we are in need of surgical attention, to whom shall we go? To the apologists for their work, to those who have operated themselves into repentance, who have made an experience only to regret it? Certainly not. Experience and judgment in this work is no more to be gotten in lumps, than can knowledge be bought by the wagonload of books.

If there has not been an antecedent experience from which special knowledge has been differentiated, this special knowledge, though it may be wide enough theoretically, practically is cramped and dwarfed. The wider the general experience, the more exact will be the special. Having considered the class of operators we must look to for errors and repentance, let us look at the work as surgery simply, not in the light of miracles it is supposed and often promised to work. Miracles nothing human can perform. No surgeon has a right to promise an absolutely certain result in every case. I have too frequently heard promised, "The operation will make you a well woman!" Patients are persuaded into operation when they should be left to choose it for themselves. I have nothing but condolence, may be contempt, for the surgeon who has to persuade his patients to be operated upon. Herein comes a great deal of the blame of surgery in the abdomen and pelvis. Who ever heard of a surgeon's having to persuade a patient to put a splint upon a broken leg or a ligature around a bleeding vessel? The indications for every operation should be plainly stated, and the patient or her responsible friends be responsible for the decision for or against operation.

The disasters of operation, on the other hand, ought not to be attributed to the inherent danger of abdominal or pelvic interference. We are to remember that, as a rule, except in extreme hemorrhage and in diseases implicating the kidneys, bladder, or ureters, abdominal operations ought to be, as a rule, successful. It is well for all aspiring operators, and for many who consider themselves established, to ponder well the words of Savage. He says:

I think we ought to get into our minds, as a prominent idea, the view that after an abdominal operation a death should be considered to an extent as preventable, and that when one does occur we should hold with ourselves a moral inquest as to the cause, how it might have been prevented, and whether in any way it was associated with aught relating to ourselves. As time goes on, I am more persuaded that in the question of success or failure, less and less depends on the patient, her conditions and surroundings, and more and more on ourselves and the attention to certain details which have been found to be essential.

With such a standard as this a man cannot fail to do the best possible work. High ideals, though they never be quite realized, are a safeguard against the nauseating complacency with which certain operators contemplate their woeful results, blaming either Providence or the nurse, allowing themselves to escape unscathed.

It is well here to consider Mr. Savage's expression when he says, "Less and less depends upon the patient, her condition, and surroundings," for this is at once too wide, and, at the same time, widely true. It is just as evident that patients go on suffering, time and again, until they are hopelessly ill, as it is that, if taken earlier, no matter what their surroundings, they could have been cured. This fact must stand out a perpetual contradiction to those who in charity advertisements for private aggrandizement praise, in season and out, the over-laudatory achievements of hospital surgery. Right here I want to say that the best results that have ever been obtained in this city, in a wide series of cases, have been done in private houses, many of them having none of the recognized conveniences either for comfort or ideal cleanliness. The room that the patient occupied was often the only clean one in the house, and yet, as a rule, all of these patients get well. We, as operators, have no business to scare a patient into a hospital for our own convenience. By doing so we bring into the chances of her recovery an additional element of doubt.

In estimating the importance of the sequelæ of any surgical operation, we must compare them with the gravity of the condition for which the operation was done. Generally speaking, promptitude in ridding a patient of any surgical disease is a step toward avoiding after-complications as well as primary impediment to rapid satisfactory work. If a patient is suffering from suppurative abdominal disease, which by its presence threatens life primarily by septicemia, or indirectly by secondary implication of vital organs, the fact that a hernia or fistula remains after operation is no argument either against the advisability of operation nor against the results of the operation. The argument rather redounds against the critics, and should convince them that early operation, while giving the patient a better primary chance for life, secondarily would leave him in better condition to resist the influences that tend to prevent prompt healing and, therefore, conduce to fistula and hernia formation. To appreciate these and other pathological points, a thorough appreciation of the pathology of pelvic inflammation is necessary. A devotee of the ancient doctrines of pelvic cellulitis has no hope of becoming either a skilful diagnostician in, or a surgeon of pelvic disease, since his supposed pathology does not agree with the actual condition of affairs; hence he is handicapped from the start, and the incubus of tradition must be fatal to his progress.

In advanced cases of tubal and ovarian disease, theory will tell him to treat the disease by derivative measures, aiding himself possibly by closure of a cervix, when lo! the patient grows worse in his hands, and is only rescued by the merest chance by final resort to an abdominal section. In such cases it is no uncommon experience to discover such a generally vicious condition that healthy tissue in which to place a ligature can scarcely be found, and the result is a fistula, through which a ligature ultimately is passed. Here, only the most careful technique, of making a good stump, cleaning out necrosed tissue for this purpose, and diligence in using accurately all precautions against infecting the ligature while it is being placed around the pedicle, will prevent the formation of fistula, or, at least, of the conditions that may result therein. Herniæ are cited as a perpetual menace against the propriety of abdominal operations. Women are said to be worse from them than from their original trouble. In these cases the value of personal opinion goes very little to announce the true condition. The surgeon or the critic of surgery who estimates the value of either medicine or surgery by the reports of the patient, will argue from very uncertain and worthless data. Some patients will complain more from a slight hernia than originally they did at the trouble necessitating operation, or at least their complaints are very distinct. In the first they groaned in anguish, begging relief, while at last they repudiate all surgery because they no longer suffer torture at each menstrual period. To this they are frequently encouraged by meddlesome women who, never having suffered, cannot appreciate the tortures of disease, or by malevolent professional rivals who descend to such indecent methods in order to compass the ruin or professional distrust of the community against the operator. As a rule, herniæ, and fistulæ especially, should be rare. Hernia is a constant danger in fat women, both as primary, owing to the uncertain healing of the fat, and secondarily as a result of weakening the abdominal support. Hence, the rule should be, in all such cases, to keep the patient in bed much longer than is required to heal the incision, and, after getting up, constantly to wear an abdominal support. A failure so to advise is just as culpable as to omit the elastic support over a dislocated patella, and can only occur as the result of sheer carelessness or ignorance of the requirements of this special class of cases. Fistulæ, besides resulting from ligatures, may have their origin in lesions of the intestine resulting from the freeing of adhesions. Fecal fistulæ are rarely persistent, almost never so, and in the great majority of cases can be avoided if a careful watch is had over the bowel involved in the adhesions. Here the result of bad work must result disastrously, both so far as perpetuating the fistula is concerned and in doing damage to the intestine. To obtain perfect results the intestine is so to be mended, not only to prevent leakage of its contents, but also to avoid adhesions compromising its function and conducing to obstruction. In this relation the deaths from intestinal obstruction after operation are to be considered. In the hands of experienced operators this rarely if ever happens, and if it occurs it is recognized and relieved. One death occurring from such cause, coming under my knowledge, was the direct result of placing the patient, shortly after operation, in the care of a physician without any experience whatever in a surgical way. The complication, accordingly, was not understood, and by the time surgical aid was sought the woman was practically dead. Imperfect after-attention of surgeons eager only to operate, has been, and will continue to be, the cause of much reproach to surgery. Until his patient is out of bed and moving about freely, the surgeon has no right to dismiss her as cured. If a hernia occur after operation, it is the duty of the surgeon to explain its nature and make early effort to cure it. The earlier it is cured the less will be the obstacles in the way of permanent relief. To have a patient die of operation for hernia, the result of the surgeon's own operation, ought to be a calamity almost unheard of. Nevertheless, it ought to be understood that, owing to the size of the sac, and the consequent extent of the adhesions, some herniæ are practically incurable unless at a great risk. One such case is still fresh in my memory, in which, after a long relief, the hernia again returned. The woman was very fat, and with the difficulties of a former operation fresh in my mind I refused another.

There is no use in operating and reoperating in unfavorable cases until at last the patient is lost. Surgery nor the surgeon receives credit, while the patient receives no benefit. Imperfect knowledge of how to drain is at the bottom of many failures in the surgery of the abdomen. I once knew the question to be asked by a man following up the matters of abdominal surgery, very far off to be sure, "How long does the tube stay in—till it smells?" I have seen a tube removed containing more than an inch of coagulated serum, this last in the hands of an operator who ought to know better than now to advise the cleaning of the tube once every twelve hours, or some such long interval. A drainage-tube that needs cleaning or emptying only once in twelve hours had better be kept clean in the bag until it is wanted. Such advice as to the use of the tube is worse than worthless. To be valuable, information must come from those who are informed, not from those who are guessing or using the tube under protest or because someone else uses it. It is better not used than badly or carelessly used. Drainage and the conditions that require it must always remain a disputed question, but one fact alone must forever keep its opponents on the defensive, and that is that those who advocate it most have had the most experience with it, and that their cases so treated run a most uneventful course, even in most serious antecedent conditions. One point negativing its advantages in some cases urgently needing it, is the semi-delirious condition of the patient during the first few days immediately following operation. These patients are unruly, and their unrest will disturb the position of the tube and render it at once irritating and useless. I have two patients in mind in whom I believe the tube on this account would have been a serious disadvantage. In another in whom I considered it advisable, I removed it at once when it had become displaced. I shall never forget the anxiety with which I watched over this patient through several days, fearing the oncoming of peritonitis and dreading the possible necessity of reopening the abdomen.

But if the proper use of the drainage-tube is essential to success, it is to be remembered that crevices created by desultory breaking up of adhesions at the bottom of the pelvis, having no common outlet by which they may drain, are beyond the reach of a single tube; hence, in appendicitis for example, the careful placing of an additional rubber tube often gives security when otherwise at the best the end would be doubtful. A case of my own is here vividly before me, and brings out the theory justified by results.

When it is sought to break up adhesions on either side of the pelvis because one side seemingly presents less difficulty than the other, the more difficult should not be abandoned when once begun, unless it is plainly evident that by freeing the simpler side a vantage-point is gained from which to attack the other. Going from side to side but gives two difficulties where but one before existed, and, in event of prolonged operation, when completion for the patient's sake must be abandoned or postponed, adds additional complications and takes away some of the chances of recovery. Such methods are common to inexperienced workers, and must be abandoned if good results are desired.

Incomplete operations are at the bottom of much of the criticism made as to the uselessness of abdominal or pelvic surgery. When an operator removes but one ovary and tube for hemorrhage of a fibroid, he confesses to the knowing critic his incompetency to deal with the conditions he meets. He is as likely to cure such hemorrhage by such surgery as to raise chickens by china eggs.

By this it becomes manifest that a lack of resource is fatal to ideal surgery. The surgeon who deserves the name is a man of emergencies. The surgeon in masquerade, like the German actor, tears not his passion but his patient to tatters. By every operation so done, a certain number of women whom surgery might save are frightened and so hindered from receiving the benefits of real surgery.

The easier operations are the bane of the would-be surgeon. Succeeding in one of these, he imagines he has conquered the whole field and at once rests easy in his assurance. To such men, and their patients generally, absolute failure in their first attempts is a distinct gain, for it frightens them away from the possibility of doing further harm.

Many other points leading to and illustrating the same idea, and showing conclusively where mal-operation and needless operation is most likely to occur, and why, could be multiplied. But this is not necessary. It is plain that that operator only is safe who has first learned by a long and painstaking apprenticeship, thoroughly and patiently, the principles of the work he is to perform; who, grounding himself in the principles, has applied them at the side of capable instructors; who, when he operates, does so for the patient's good and not for his own glory. Such a man—such men—must both give their patients their best hope and be the saviours of surgery from its false exponents, who only disgrace it.

The men who get the best results are those who work along safe lines, departing from them as necessity compels according to the exigencies of each individual case; not those who from the threading of a needle to the cleansing of a tube or the washing of their hands strive to be original. Such originality hides real surgery in a multiplicity of details and paraphernalia, and risks the sufferer to exalt the operator.

DISCUSSION.

Dr. M. PRICE: I am fully in accord with the belief that most of our deaths can be accounted for by some want of care on the part of the operator before, during, or after operation. I recall two cases in which the previous care before operation had everything to do with the result. In one, the woman was sent into the house to be operated on. The patient declared that the bowels had been freely moved fifteen or sixteen times on that day. The operation was done, and it was found that the patient had not had the bowels opened for two weeks. She had an impermeable stricture, which would permit the passage of nothing but the watery contents of the bowel. The fatal result might have been avoided if the patient had been under the care of a trained nurse before the operation. I have seen this accident twice.

Many men who profess to be abdominal surgeons will go to a case of supposed tumor, or some other condition, prepared to do only that operation. They go to do an ovariotomy, and a hysterectomy is required. The result is a death. The surgeon must carry with him everything that can be needed. I have seen many hundreds of abdominal sections, and have seen about as many mistakes as most men, and that, too, in the most competent hands.

As to the success of abdominal surgery: Does anyone pretend to say that when a surgeon amputates a leg, giving the patient a useful stump, that is not a success? I hold that it is. If we had subjected an abdominal case to such a risk of life, and had left it in such a crippled condition, what a howl there would be on the part of the medical profession! You take a woman who has been lying around, unable to assist herself in the slightest, and you remove the disease and allow her to move around with some little pain, some little crippling of her gait. You cannot expect to make a perfect cure. Many of the objections, and many of what are called sequelæ, are the fault, not of the surgeon, but of the patient's delay.

Dr. G. BETTON MASSEY: The attempt has apparently been made to make an apology for surgery. I think that from some points of view the surgery of the abdominal cavity needs an apology. By some we are told that the bad results are due to improper operations, and by others that they are due to the diseases themselves. I would take no exception to that class of bad results in those cases of abdominal disease incapable of being cured in any other way than by surgery. I have been much impressed with the mistake which is the keynote of Dr. Hoffman's paper—that is, the mistake made by surgeons of persuading women to be operated on who do not require any operation. I have seen a number of these patients who, fortunately, have failed to take the advice given by the surgeon. I have now under observation a lady who was persuaded to have her ovaries removed in order that her cervix might be repaired. A little reduction of the congestion and enlargement of the parts in a rather stout patient showed that the whole trouble came from a subinvoluted uterus and a hyperplastic enlargement of one side, which was rapidly improved.

Dr. O. H. Allis: It seems to me that a good deal depends upon who gives the advice whether an operation should be done or not. A patient comes with sarcoma of the jaw, where nothing but removal of the entire jaw, or at least half, will save life. Although the surgeon urges operation with earnestness, the patient goes away and nothing is done. The delay is fatal. The same is true in strangulated hernia. The patient should be urged to undergo immediate operation.

Dr. William E. Ashton: This question of hernia following abdominal section is one of great importance. The surgeon who says that he never has a hernia does not follow up his cases. The majority of hernias are due to a failure in properly uniting the sheath of the rectus. I do not believe that fat women are especially liable to this accident. The integrity of the abdominal wall depends entirely upon the sheath of the rectus muscle, and this should be sutured in all cases. I believe, also, that a large number of hernias are due to getting out of bed too soon. The patient should not get up under three weeks. There is not much benefit in an abdominal support. I have found it impossible to keep any bandage closely enough applied to relieve the ordinary strain. If the patient is doing heavy work, an abdominal support may be of service.

In regard to fistula caused by a ligature, I might mention a clever device told to me by a doctor from Texas. He takes a number of horse-hairs, doubles them over upon themselves and ties them together, and passes them into the fistula and turns them around. In this way he often succeeds in bringing away the ligature.

I believe it to be a good plan to have the patient in a hospital, if possible, in order that she may be under observation and control. I do not believe that there is more danger of sepsis in cases operated on at home than in those in hospitals. Sepsis does not come from the atmosphere. Barring the question of control, there is little difference, so far as results are concerned, where we operate.

Dr. J. Price: Dr. Allis has given an interesting point in reference to the selection of cases and the importance of promptitude. The whole experience of abdominal surgeons is in favor of promptitude in pelvic abscess and in small tumors of all kinds. I may say in regard to mortality, that if my mortality equaled the number of cases that I find dead when I arrive at the house, I should cease to operate. A man to do abdominal surgery should serve an apprenticeship, not only that he may know what to recognize, but that he may, with specimens in hand, compare them with the histories. With a pair of pus tubes or a pelvic abscess, you can almost demonstrate the number of attacks of pelvic peritonitis that the woman has suffered.

It is all very nice to talk about the sequelæ of abdominal sections, but the sequelæ antedate the operation in many cases. I saw, today, a large cystoma, probably complicated with fibroid. The patient had been counseled by many not to have the tumor removed. I sometimes wish that the general practitioner and general surgeon were like the eye and ear men—they attend to their business and say nothing about anything else.

Dr. J. M. Baldy: Unquestionably many of the so-called sequelæ occur before the section has been performed. Many, also, are due to incomplete operations. I do not think that any one cause is at fault. I have not found that fistula and hernia are more apt to occur in fat women, nor have I found that suturing of the fascia will prevent it.

In regard to hospitals, I believe in railroading patients into hospitals if you have well-appointed ones. You have there a better chance to do good work and to watch the patient. Some of my best work, however, has been "alley work," but it has been most anxious work. In these cases I have been forced to permit them to get up in two weeks. In hospitals you can keep the patient in bed. I think that four weeks is not too long.

As to the cause of death, I have seen one death from shock in the hands of another gentleman. I have seen one or two deaths from hemorrhage, but the one great cause of death is septic peritonitis. In some of these cases I have thought that the tube was at fault, but in others there was no drainage. In regard to early operation, if it is an operative case, it cannot be done too soon.

Dr. Charles P. Noble: In regard to the cause of death after abdominal section, I may say that in my own experience the deaths, with two exceptions, were due to the fact that the patient was operated on when she was almost dead from disease. I have had three deaths from kidney trouble. In two the condition was not discovered prior to operation. Serious kidney disease is a positive contra-indication to abdominal section involving any considerable manipulation.

I think that our facilities for managing patients are much better in hospitals than at home, and I think that there is every inducement, both on account of the patient and ourselves, to urge her to go to a hospital.

In reference to cellulitis. Most men who do abdominal surgery say that they never see cellulitis. I have seen it but do not think that it is a common condition. I do not believe that we meet with it as a non-puerperal condition. Cellulitis in the pelvis occurs as does cellulitis in other parts of the body—from infection. I have knowledge of four abdominal operations in which the tubes were examined and found free from pus accumulation. Of course, it was not possible to say that catarrhal salpingitis was not present. Large quantities of pus, however, were evacuated through an incision over the ramus of the pubes. They were undoubted cases of pelvic abscess. I believe that this accounts for the fact that certain women recover and bear children after they have had pelvic inflammation complicating labor.

In regard to drainage : it has become my practice to drain in almost every case, and I have seen no reason to regret it. Unless there is a special reason to the contrary, I drain every case. It is often said that unless the tube is cleaned every half hour to two hours, the surgeon does not understand drainage. At the Kensington Hospital for Women, there has been a series of as many as sixty cases, with one death, where this principle of drainage was not used. In the last thirty-three cases (without a death), the drainage-tube was not evacuated oftener than three times in twenty-four hours. My experience with the capillary drain, running over one hundred cases, enables me to say that it is a most efficient form of drainage, and that it is the exception, even in bad cases, to find more than a drachm of fluid in the pelvis when the tube is drained. By this measure the pelvis is continually drained.

Dr. Hoffman : While a capillary drain will remove the liquid, it will not remove clots. The syringe will. All that my point stated was that cleaning the drainage-tube once in twelve hours is *not* cleaning it.

In regard to hospitals, I can control my patients better in their homes. The hospital is not a *sine qua non*, and is not free from danger.

In speaking of advice in surgery, I did not object to real advice. We, however, compromise ourselves by seeking operations, although those things are done. If an operation is necessary, we should say so, and tell why it is. If the patient does not accept it, the result does not lie at our door.

